# Homogeneity among glyphosate-resistant *Amaranthus palmeri* in geographically distant locations

**DOI:** 10.1371/journal.pone.0233813

**Published:** 2020-09-09

**Authors:** William T. Molin, Eric L. Patterson, Christopher A. Saski

**Affiliations:** 1 Crop Production Systems Research Unit, United States Department of Agriculture, Stoneville, Mississippi, United States of America; 2 Department of Plant, Soil, and Microbial Sciences, Michigan State University, East Lansing, Michigan, United States of America; 3 Department of Plant and Environmental Sciences, Clemson University, Clemson, South Carolina, United States of America; University of Illinois at Urbana-Champaign, UNITED STATES

## Abstract

Since the initial report of glyphosate-resistant (GR) *Amaranthus palmeri* S. Watson in 2006, resistant populations have been reported in 28 states. The mechanism of resistance is amplification of a 399-kb extrachromosomal circular DNA, called the *EPSPS* replicon, and is unique to glyphosate-resistant plants. The replicon contains a single copy of the 10-kb 5-enolpyruvylshikimate-3-phosphate synthase (*EPSPS*) gene which causes the concomitant increased expression of EPSP synthase, the target enzyme of glyphosate. It is not known whether the resistance by this amplification mechanism evolved once and then spread across the country or evolved independently in several locations. To compare genomic representation and variation across the *EPSPS* replicon, whole genome shotgun sequencing (WGS) and mapping of sequences from both GR and susceptible (GS) biotypes to the replicon consensus sequence was performed. Sampling of GR biotypes from AZ, KS, GA, MD and DE and GS biotypes from AZ, KS and GA revealed complete contiguity and deep representation with sequences from GR plants, but lack of homogeneity and contiguity with breaks in coverage were observed with sequences from GS biotypes. The high sequence conservation among GR biotypes with very few polymorphisms which were widely distributed across the USA further supports the hypothesis that glyphosate resistance most likely originated from a single population. We show that the replicon from different populations was unique to GR plants and had similar levels of amplification.

## Introduction

In 2006, glyphosate resistant (GR) *A*. *palmeri* S. Watson was reported in Georgia (GA) [1], and since then, glyphosate-resistant populations have spread to 27 other states as far west as California, and as far north as Maryland (MD) and Wisconsin [2]. The resistance mechanism in *A*. *palmeri* was identified as amplification and seemingly random insertion of the 5-enolpyruvylshikimate-3-phosphate synthase (*EPSPS*) gene into the genome [3]. *EPSPS* gene sequences from GR and GS plants were identical indicating the resistance was not due to a point mutation. GR biotypes of *A*. *palmeri* typically contained between 40 and 100 copies of the *EPSPS* gene per genome equivalent [3–7] which increased the C-value of the genome by up to 11% in resistant plants [5]. Interestingly, glyphosate resistance by copy number variation and increased genome size does not seem to lead to immediate consequences to overall fitness [8–10]. The long-term evolutionary consequences of increases in DNA content from targeted amplifications are unknown and may be largely dependent on the level of expression inherited by future generations.

Previous work demonstrated that the amplified *EPSPS* gene was part of a co-amplified 297-kb sequence, the so-called the *EPSPS* cassette [5]. Polymerase chain reaction products from 40 primer pairs distributed equidistantly along this amplified, 297-kb sequence were nearly identical in size and sequence to those in other resistant populations from Arizona (AZ), Kansas (KS), Maryland (MD), Delaware (DE) and Georgia (GA) [6]. However, many of the primer pairs failed to produce products with DNA of GS plants from AZ, KS and GA [6]. These results supported the hypothesis that resistance evolved once, and then spread rapidly by natural means and/or human intervention. However, the study was inconclusive because the primer pairs did not produce overlapping sequences, and therefore were not contiguous, along the 297-kb *EPSPS* cassette [5].

We recently reported that the 297-kb cassette was part of extrachromosomal circular DNA named the eccDNA replicon [11]. The sequence and reference assembly of this eccDNA indicated it was a massive 399,435 bp episome-like DNA containing 59 predicted coding gene sequences, including the *EPSPS* gene, 41 of which were also transcribed [11]. In addition to the *EPSPS*, other genes include multiple AC transposases, replication proteins, a reverse transcriptase, heat shock, and other genes with unknown function [11]. The eccDNA replicon was composed of sophisticated repetitive arrays and was heavily punctuated with sharp changes in A+T and G+C content. It was also found that orthologous genes encoded in the replicon were not found in a colinear organization when aligned to the chromosome-scale reference assembly *A*. *hypochondriacus* and *A*. *tuberculatus* [11] which indicates that the eccDNA replicon was assembled from distal parts of the genome instead of a focal amplication of a local genomic segment surrounding the *EPSPS* gene. In a few limited reports, stable, extrachromosomal linear and circular DNAs have been found in plants [12, 13], and the *EPSPS* eccDNA replicon probably represents a novel extension of these. In addition, high-resolution fiber-FISH analysis provided cytological resolution and evidence that the *EPSPS* replicon exists outside the chromosomes in circular forms [14]. In this work, six overlapping BAC tile path probes showed a circular arrangement that includes the *EPSPS* replicon in the GR biotype from MS [14]. In addition to free, extra nuclear circular forms, this eccDNA was also found as chromosomally integrated linear forms (~30%) and as circular forms attached to metaphase chromosomes through a putative tethering mechanism indicating that the eccDNA is heritable [14]. Unequal segregation of extrachromosomal replicons due to unequal tethering to chromosomes at cell division may account for the nonmendelian inheritance patterns observed in F_2_ populations [15–19].

Whether the *EPSPS* replicon has single or multiple origins is a pertinent question that impacts both our understanding of resistance evolution, as well as the practical task of GR *A*. *palmeri* management. A previous study used genotyping-by-sequencing to measure the relatedness of geographically distant, glyphosate resistant populations, however, they were unable to draw a strong conclusion due, in part, to the high amounts of genetic variation in the diploid genome between individuals [20]. *A*. *palmeri* is dioecious and morphologically diverse, and as such, *EPSPS* replicon sequence diversity may be expected among resistant populations from geographically distant locations, especially if the replicon evolved independently in divergent populations. To further characterize the similarity among the *EPSPS* replicon sequences from divergent GR *A*. *palmeri* lines and gain evidence for or against a common origin from a single source, a whole genome approach was taken to characterize sequence variation in the entire *EPSPS* replicon as well as genome-wide gene copy number variations in *EPSPS* and other genes in the replicon. In this study, whole genome shotgun sequencing was conducted on GR plants from AZ, GA, KS, DE, and MD and GS plants from AZ, GA and KS, to characterize presence and variation of the eccDNA replicon reference sequence from Mississippi. We show that the *EPSPS* replicon is highly conserved across vast distances in the USA.

## Materials and methods

### Plant sources, resistance confirmation, growth conditions

Seeds were collected from individual GR plants that had survived glyphosate application as previously described [5]. *EPSPS* copy numbers were determined by qPCR on leaves from the third and fourth nodes from two plants from each seedling sample. Copy number assays were performed multiple times as indicated (Table 1). Single populations from other states were provided by M. Jugulam, Kansas State University, W. McCloskey, University of Arizona, B. Hoagland, USDA-ARS, and M. VanGessel, University of Delaware.

**Table 1:** *EPSPS* copy numbers for the eight biotypes, five GR and 3 GS, used herein by qPCR compared to the copy number variation (CNV) determined by the CNVnator program.

Origin	Glyphosate sensitivity	Average Copy Number	Number of DNA isolations per seed source	CNVnator estimate of read depth
Arizona	Resistant	77	4	279
Arizona	Sensitive	1	4	0.4
Georgia	Resistant	35	2	177
Georgia	Sensitive	1	2	2.5
Kansas	Resistant	69	4	156
Kansas	Sensitive	2	4	2.8
Maryland	Resistant	35	3	271
Delaware	Resistant	38	3	238
Mississippi-13	Resistant	38	12	—

Plants were grown in 9 × 9 × 9 cm plastic pots that contained a commercial potting mix (Metro-Mix 360; Sun Gro Horticulture, Bellevue, WA, USA). Seeds were sown on the potting mix surface and lightly covered with 2 mm of potting mix. Pots were sub-irrigated and maintained in a greenhouse set at a temperature regime of 30/25 ^∘^C (day/night) and a 15-h photoperiod under natural sunlight conditions supplemented with high-pressure sodium lights providing 400 μmol m^−2^ s^−1^. Sampling for whole genome sequencing was performed using a leaf from the third node of two representative plants from each population.

### qPCR determination of *EPSPS* copy number

qPCR was performed to determine copy numbers of *EPSPS* in plants from the eight plant sources using primer pairs CAACAGTTGAGGAAGGATCTG (AW146) and CAGCAAGAGGAAGGATCTG (AW147) [6]. Acetolactate synthase gene (*ALS*) was used as a housing keeping gene with primers GCTGCTGAAGGCCTACGCT (AW23) and GCGGGACTGAGTCAAGAAGTG (AW24) [6]. DNA was extracted from two leaves each from different seedlings. Reactions consisted of 10 ng of genomic DNA,1.5 μM primers, 1 X Power Sybr Green master mix (Life Technologies), and H2O to 50 μL. Reactions were performed using an ABI 7500 Real Time PCR System. Cycle conditions are as follows: 50°C for 2 min, 95°C for 10 min, 40 cycles of 95°C for 15 s and 60°C for 1 min, and a 4°C hold. A melt curve analysis was included. Data were analyzed according to the standard curve method and error was calculated.

### Whole genome shotgun re-sequencing and variant discovery

Whole genome shotgun sequencing of 5 GR (AZ-R, KS-R, GA-R, DE-R, MD-R) and 3 GS (KS-S, GA-S, AZ-S) biotypes was performed on total genomic DNA isolated by standard CTAB plant DNA extraction procedures as previously described [6]. DNA was extracted from two leaves each from different seedlings. Sequencing libraries were prepared by ultrasonic shearing of the gDNA with a Covaris ultrasonicator (Covaris) to an average size of 500-bp and indexing with sequencing adapters using the TruSeq DNA (PCR free) kit (Illumina). Sequencing was performed on a single lane of an Illumina HiSeq2500 with 500 cycles in high-output mode. Raw data was preprocessed to remove adapter and low-quality sequences with the Trimmomatic software [21], and cleaned reads mapped to the reference *EPSPS* replicon sequence with the Bowtie2 short read mapper using default parameters [22]. Reads that did not map to the eccDNA reference sequence were subset to a file. BAM alignment files for the eccDNA replicon were filtered for concordant read alignments and mapping quality with the Samtools toolset (v1.3.1) and read depths were determined with the Samtools ‘depth’ function [23]. Single nucleotide variants were determined with the HaplotypeCaller walker of the GATK v3.5 software package and outputted in variant call format (VCF) [24]. Variant sites were filtered for depth (DP>10) and mapping quality (MQ>30) with vcftools [24]. Reads that did not map the eccDNA replicon were aligned to the glyphosate sensitive reference assembly (SAMN14120689) with Bowtie2 short read mapper using default parameters [22]. Single nucleotide variants were determined with the HaplotypeCaller walker of the GATK v3.5 software package and outputted in variant call format (VCF) [24]. A pruned set of SNPs that are in approximate linkage equilibrium were subset with the snpgdsLDpruning function of SNPRELATE v.1.22.0 and an LD threshold of 0.5. Hierarchal clustering using principal components was performed on the LD pruned SNPs with SNPRELATE v.1.22.0 and plotted in R. Circular figures were plotted with the Circos plotting tool [25]. Cleaned reads were aligned to the entire AZ-S genome assembly modified by concatenating with the entire replicon sequence using Bowtie2 short read mapper [22]. BAM alignment files were filtered for concordant read alignments and mapping quality with the Samtools toolset [23]. These BAM files were then analyzed for copy number variants (CNV) using CNVnator [26, 27] using standard parameters and a bin size of 500bp. The outputs were then turned into BED files and intersected with the annotation BED file for the whole genome using BedTools [28] to call copy number variants for every gene in the genome and the replicon.

### *Amaranthus palmeri* (GS) genome, *EPSPS* reference sequence, and WGS availability

The *Amaranthus palmer* (GS) reference sequence for this article can be found in the EMBL/GenBank data under BioProject ID PRJNA606296; Submission GenBank SAMN14120689. The *EPSPS* reference sequence (GR) for this article can be found in the EMBL/GenBank data under BioProject ID PRJNA413471; Submission GenBank MT025716. Whole genome resequencing datasets for each state and biotype used in this study can be found in the EMBL/GenBank data under BioProject ID PRJNA413471; Submission Genbank SAMN13521421-SAMN13521428.

## Results and discussion

The *EPSPS* gene copy numbers were determined for the five GR biotypes and three GS biotypes and are shown in Table 1. The standardized read depth for every gene in the eight *A*. *palmeri* whole genome resequencing datasets was calculated by CNVnator with a sliding 500bp window and is presented in S1 Table. Relative copy number of EPSPS determined by qPCR was proportional with relative read-depth of the replicon as determined by the software CNVnator.

**Fig 1:**
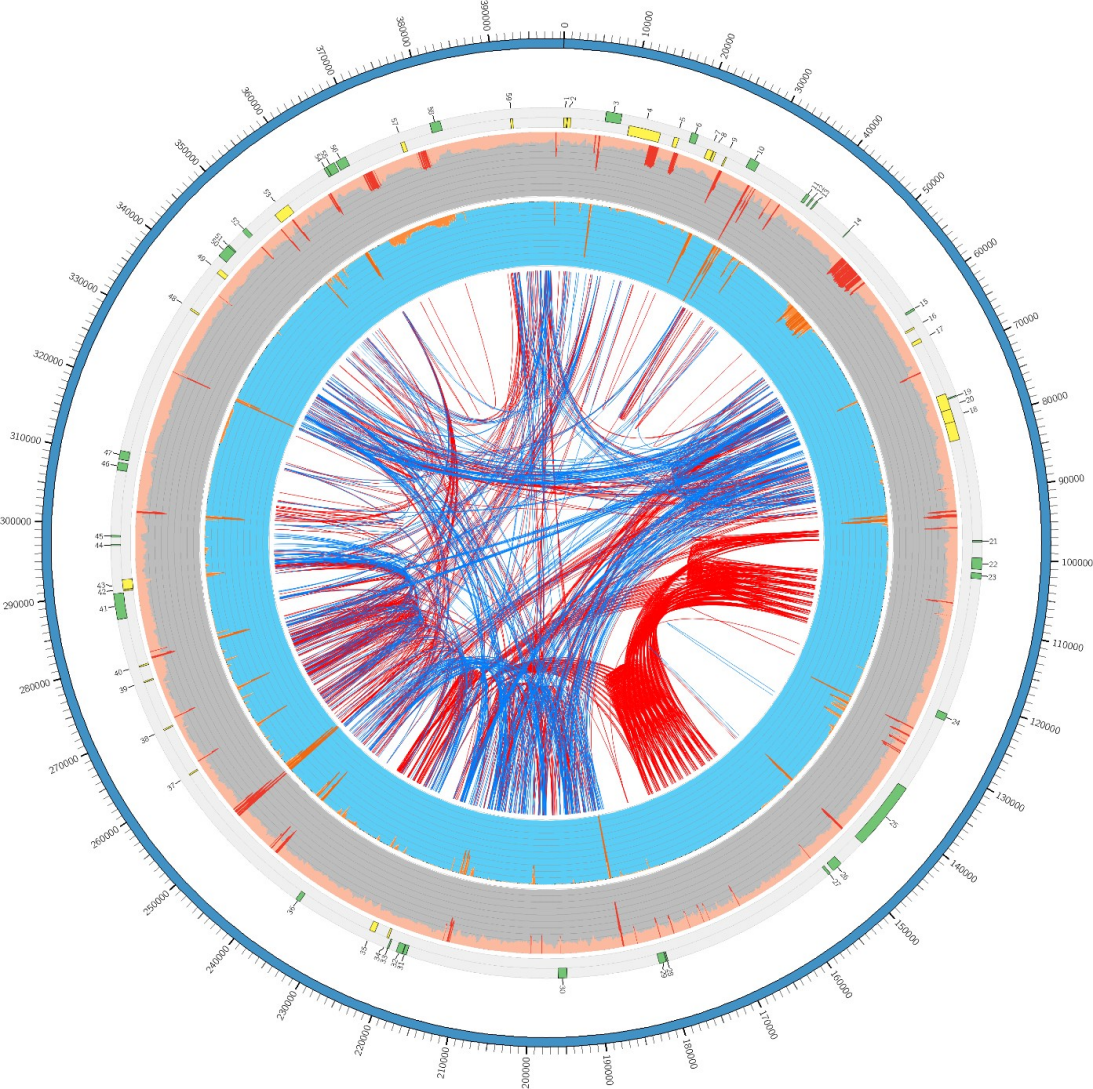
The 399,435 kb *EPSPS* replicon. Whole genome shotgun resequencing of glyphosate resistant and susceptible biotypes and mapping of reads of both biotypes (GR and GS) to the reference *EPSPS* replicon was performed. The outer ideogram (blue) is the 399, 435 kb reference assembly. The outer gray and inner bright blue tracks are circular graphs, the diameter of which represents 12000 WGS reads. The light pink band along the outer edge of the gray track is the read depth for a GR plant and indicates a read depth about 2000 whereas a similar continuous track is not present from GS plants. The inner bright blue track represents GS WGS coverage. In the gray background, light pink (GR) colors, and dark red mark regions covered by greater than 2,001 reads. Read depths for GS biotypes are orange. Interestingly, WGS depths for GR were congruent in depth across the *EPSPS* replicon; while the GS WGS data contained gaps. The network of red and blue line in the center of the figure represent connect identical sequences of tandem repeats (red) and indirect repeats (blue).

**Fig 2:**
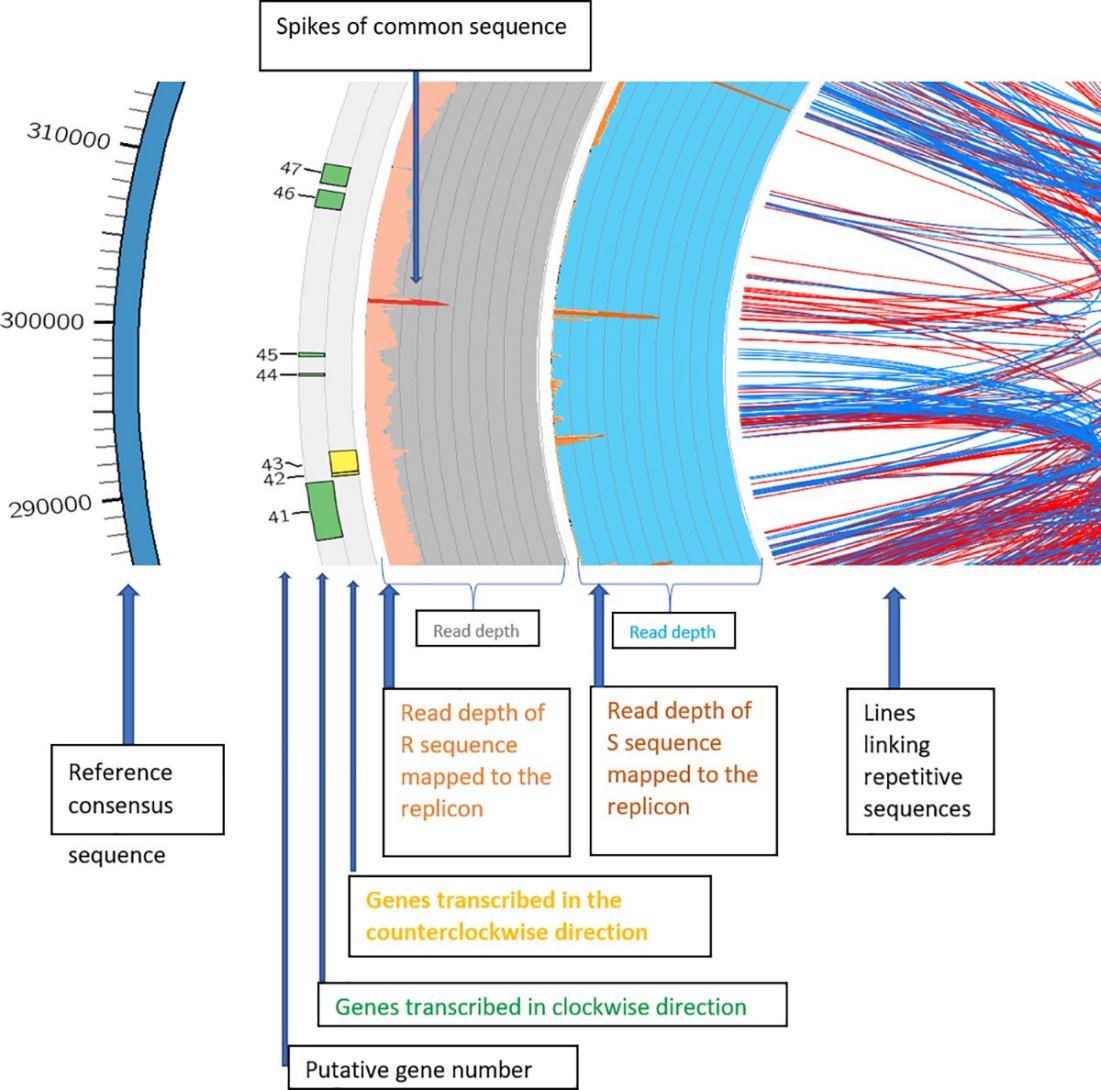
Enlargement of a segment of Fig 1. 300 kb showing a red/orange-colored spike representing regions of high copy numbers relative to the rest of the genome that has been acquired by the *EPSPS* replicon. The light pink band along the outer edge of the gray track is the read depth for a GR plant and indicates a read depth about 2000 whereas a similar continuous track is not present from sensitive plants.

In order to analyze *EPSPS* replicon representation, variation, and configuration, sequence reads were aligned as pairs to the *EPSPS* consensus sequence, which contains a single copy of the *EPSPS* gene flanked by 135-kb of sequence upstream and 244-kb downstream (Fig 1). The outer blue ring represents the consensus sequence of the 399,435 bp contig. Consecutively numbered gene annotations are presented as histograms in the light gray inner track (*EPSPS* is gene 25 on the replicon) and the direction of transcription of putative transcribed genes is indicated by the color exterior and interior of the center axis of the light gray band, eg. green and exterior is clockwise and yellow and interior is counterclockwise (Fig 1). The next two broad, concentric inner rings, gray and blue, represent the results of WGS sequencing and mapping of read pairs from GR and GS samples from Mississippi to the *EPSPS* consensus sequence. This approach provided a means to assess representation, contiguity, and variation between the sensitive and resistant biotypes (Fig 1). The gray and blue concentric rings are graphic representations of read depths up to ~8,000 reads covering certain bases. The light orange glyph (along outer edge of gray circle) against the gray background depicts read depths from GR plants. Dark orange indicates bases that are covered by greater than 2,000 reads. The glyphs along the outer edge of the blue track are incomplete indicating gaps in sequence for reads from sensitive plants. The read depth in GR confirmed the presence of a contiguous circular replicon sequence and the lack of contiguous sequence in GS (Fig 1). The red/orange spikes in both tracks are areas of higher read depth represent sequences common to both genomes, possibly originating from low-complexity sequence, repetitive elements, or other high copy number DNAs present in higher abundance in the genome (Figs 1 and 2). These spikes may indicate that the replicon is a re-arranged assembly of the GS genome because of the lack of similar read pair alignments from the GS WGS datasets.

The central core of Fig 1 shows red and blue links of repetitive sequences. The *EPSPS* gene was surrounded by dense concentrations of tandem repeats (indicated by the red line links) which align to putative gene sequences that were not found clustered in S plants (Fig 1). The tandem repeats flanked the *EPSPS* gene, but largely excluded the inverted repeats (indicated with blue links in the center). In the GS WGS sequences, these repeat regions are not present at the same abundance in comparison to other regions of the eccDNA replicon, suggesting they are lower in abundance in the GS genome.

**Fig 3:**
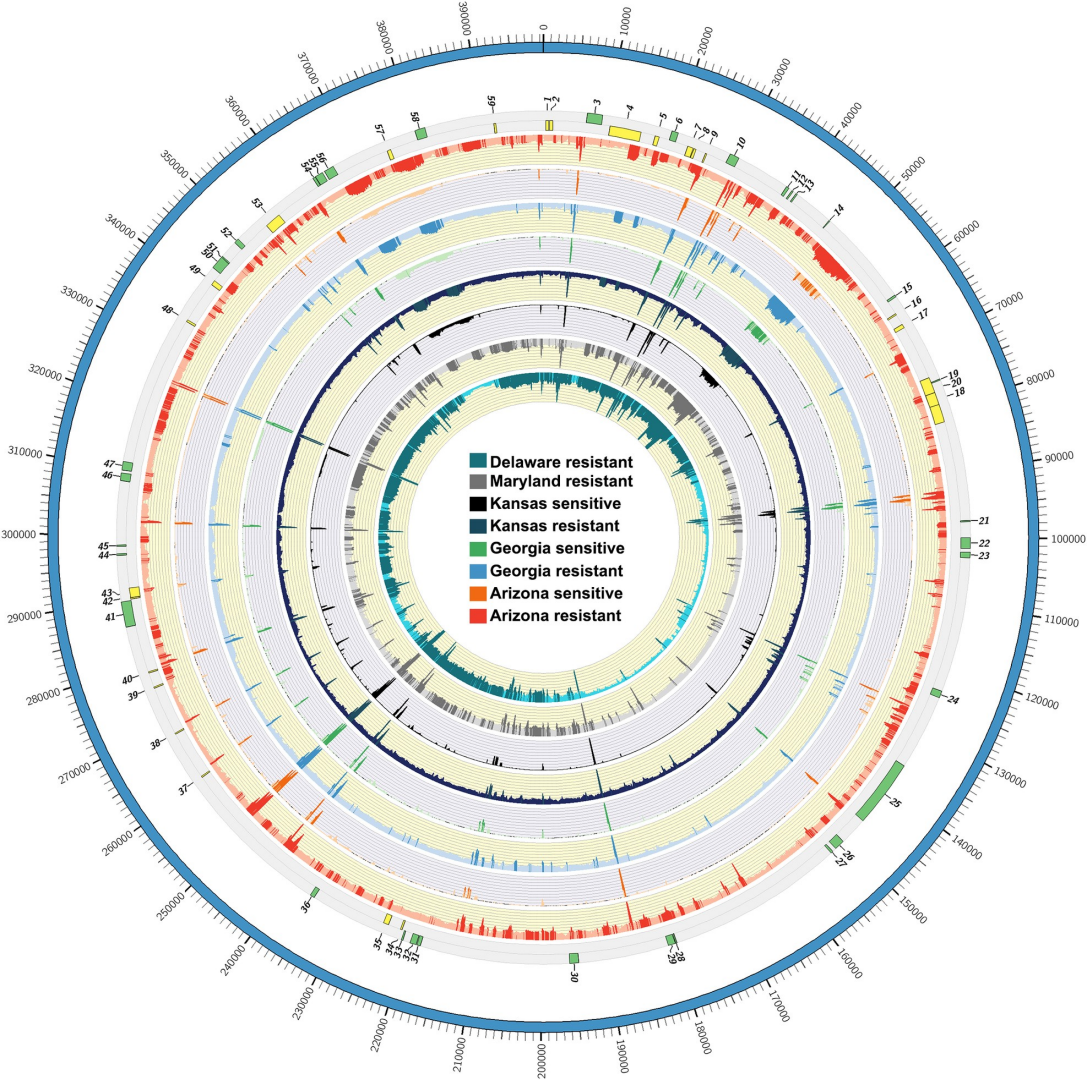
Mapping and alignment of WGS reads from R and S plants (a) Mapping and alignment of WGS reads from GR and GS plants from different locations and populations from across the USA to the *EPSPS* replicon.

**Fig 4:**
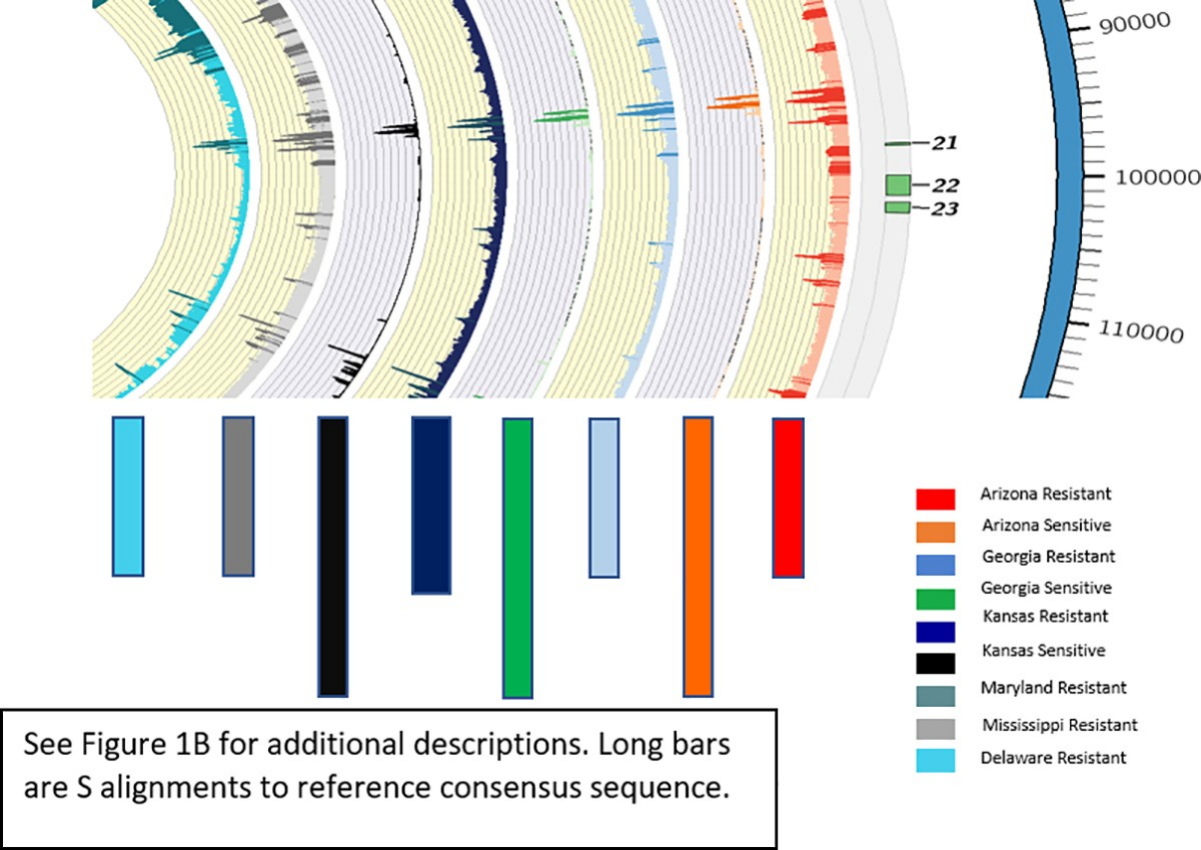
Enlarged segment of Fig 3. From left to right, short arrows point to color matched read depths of GR plants from Delaware (DE-R), Maryland (MD-R), Kansas (KS-R), Georgia (GA-R), and Arizona (AZ-R aligned to the Mississippi reference assembly and long arrows point to color matched read depth of GS plants from Kansas (KS-S), Georgia (GA_S) and Arizona (AZ_S) alignments.

WGS sequencing was conducted on GR biotypes from five states (AZ, GA, KS, DE and MD) and GS biotypes from three states (AZ, GA, KS). An average of 58 million read pairs per sample (approximately 36X genome coverage based on 410 Mbp haploid genome size of the glyphosate sensitive genome) were collected. Read pairs were mapped to the *EPSPS* replicon consensus sequence to assess representation, contiguity, and variation between the GR and GS biotypes (Figs 3 and 4 and S1 Table). Greatly contrasting alignment rates were found after filtering for concordant pair alignments and quality (Q>20, Table 1). Each GR biotype mapped at least 2.2 M read pairs, with greater than 92% aligning in proper pairs (Fig 1). Conversely, the WGS reads derived from GS biotypes had less than 200 k high quality aligned pairs, where 50% or less were in the proper orientation (Figs 3 and 4). Average read mapping depths of GS biotype sequences to the replicon varied by a magnitude of 5. All GR biotypes had at least 1,000 reads mapped when averaged; while all GS biotypes had no more than 181 reads as an average depth (S1 Table). When Illumina data from GS biotypes was aligned to the GR replicon, there was a striking lack of sequence contiguity; furthermore, many regions of the GR replicon had no Illumina data that aligned to it suggesting those regions were missing from the sampled GS biotypes (S1 and S3 Tables). Therefore, the results further support the conclusion that the *EPSPS* replicon is not present in its entirety in the background of the *A*. *palmeri* genome and was somehow assembled from many pieces from distal parts of the genome.

The WGS reads from the three GS and five GR biotypes were aligned against the whole-genome assembly of a susceptible individual from the AZ-S population with the addition of the full-length replicon. These alignments were analyzed using CNVnator for the detection of other putative CNV events and/or replicons. CNVnator detected several putative duplications and deletions, most with relatively small changes in read depth (S2 Table). Some CNV events indicated extremely high amounts of increased read depth; however, besides the EPSPS replicon, no other copy number variant was detected that distinguished GR from GS biotypes, i.e. that was unique in all GR and not GS or *vice versa*. The read depth and relative copy number of all 59 genes in the GR replicon was homogenous, indicating that the replicon does not exist in multiple configurations either within an individual or across individuals from distant geographic regions. Some putative CNV events were biotype specific or regionally specific (i.e. KS, GA, AZ, MD, DE). These were generally few and did not show drastic deviation from background read depth. There were also several putative CNVs detected in all eight biotypes, indicating the genome was incomplete and did not accurately represent highly repetitive elements. (S2 Table).

The question of whether the *EPSPS* replicon evolved once and rapidly moved across the USA or evolved many times in different locations was addressed by assessing single nucleotide polymorphism (SNP) variation of the GR and GS biotypes from geographic distant locations from across the USA relative to the *EPSPS* replicon from Mississippi. Within the 399 kb sequence, a total of 5,079 SNPs and 738 Insertion/Deletions (INDELS) were identified among the variant sites that were genotyped (Table 2 and S3 Table). Consistently, the GS biotype samples contained approximately 4-fold more SNPs than the GR biotypes; while GR biotypes had very few private SNPs (<10), except for the Maryland sample which had only 277 private SNPs (Table 2).

**Table 2:** Genotyping statistics from WGS reads relative to the *EPSPS* replicon of GR *A*. *palmeri* from Mississippi.

State	Biotype	SNP Count	Indel Count	Private	Missing
Arizona	Resistant	323	113	3	65
Arizona	Sensitive	2359	289	946	2162
Delaware	Resistant	495	166	10	48
Georgia	Resistant	465	133	4	65
Georgia	Sensitive	1858	133	413	2881
Kansas	Resistant	458	125	2	74
Kansas	Sensitive	2022	247	497	2203
Maryland	Resistant	945	179	277	380

**Fig 5:**
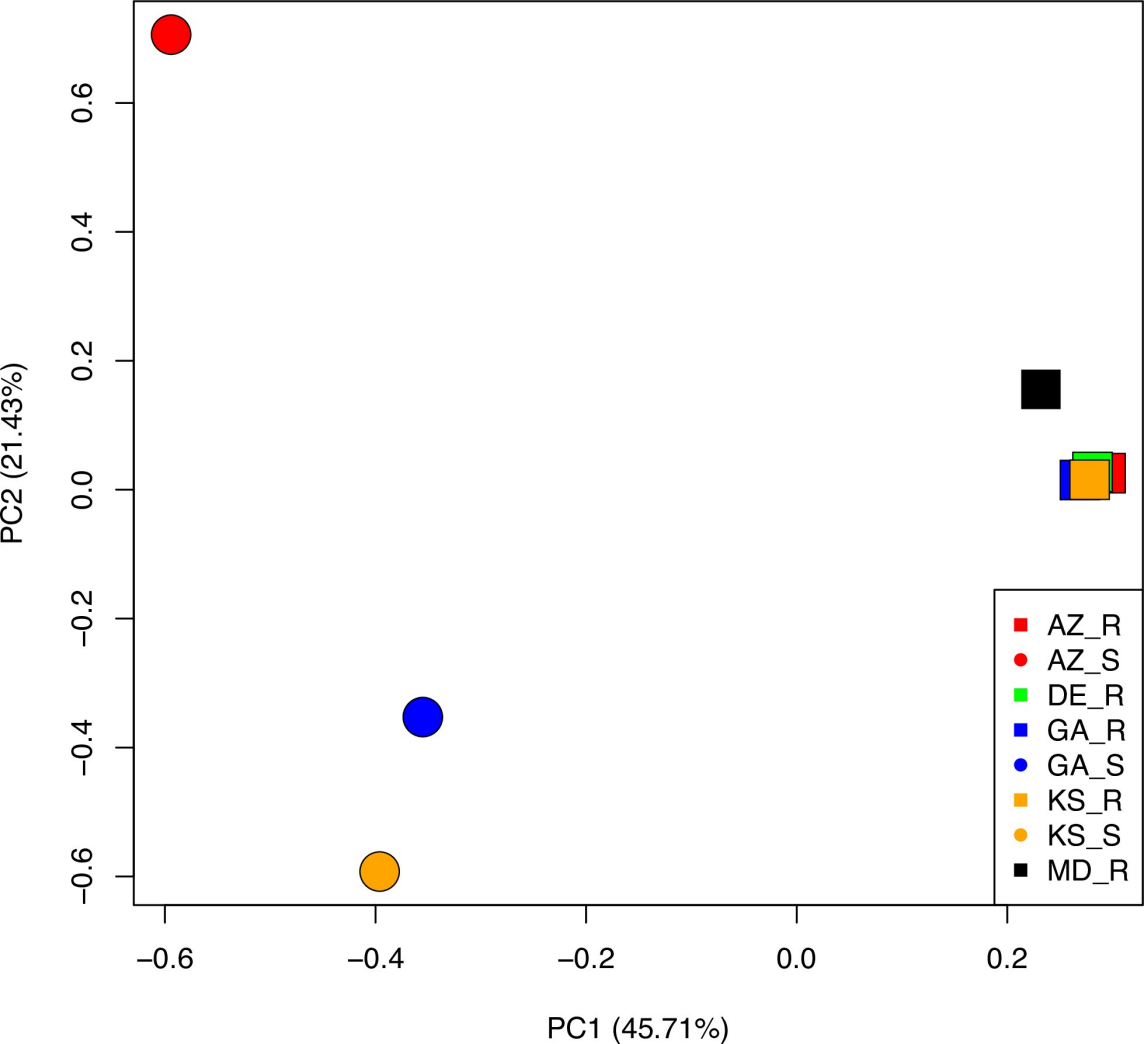
Principal component analysis of WGS reads from R and S plants. Principal component analysis of WGS reads from GR and GS plants from different locations indicating the level of similarity among *EPSPS* replicons. Note that the symbol for GA_R is nearly covered by that of KS_R.

Clustering of the samples based on SNPs revealed that the GR biotypes formed a distinct group from the GS biotypes (Fig 5). The GR biotypes were tightly clustered with only Maryland (MD-R) appearing as a slight outlier to the other GR biotypes. The GS biotypes were widely separated from the GR biotypes and from each other by the first and second principal components. The sequences from the different GR biotypes were also had similar sequence representation. Points along the replicon having greater read depths, which appear as spikes (Figs 3 and 4), also aligned perfectly with each other. Not only did these WGS datasets have highly conserved sequence, the contiguity among the genes and repetitive sequences along the length of the replicon was preserved in GR but not GS biotypes. Alignment of WGS reads from GS biotypes to the *EPSPS* replicon revealed gaps in the sequence in all the sensitive populations tested, and very low sequence coverage for the *EPSPS* replicon genes that are co-amplified in GR biotypes, as previously described [5].

**Fig 6:**
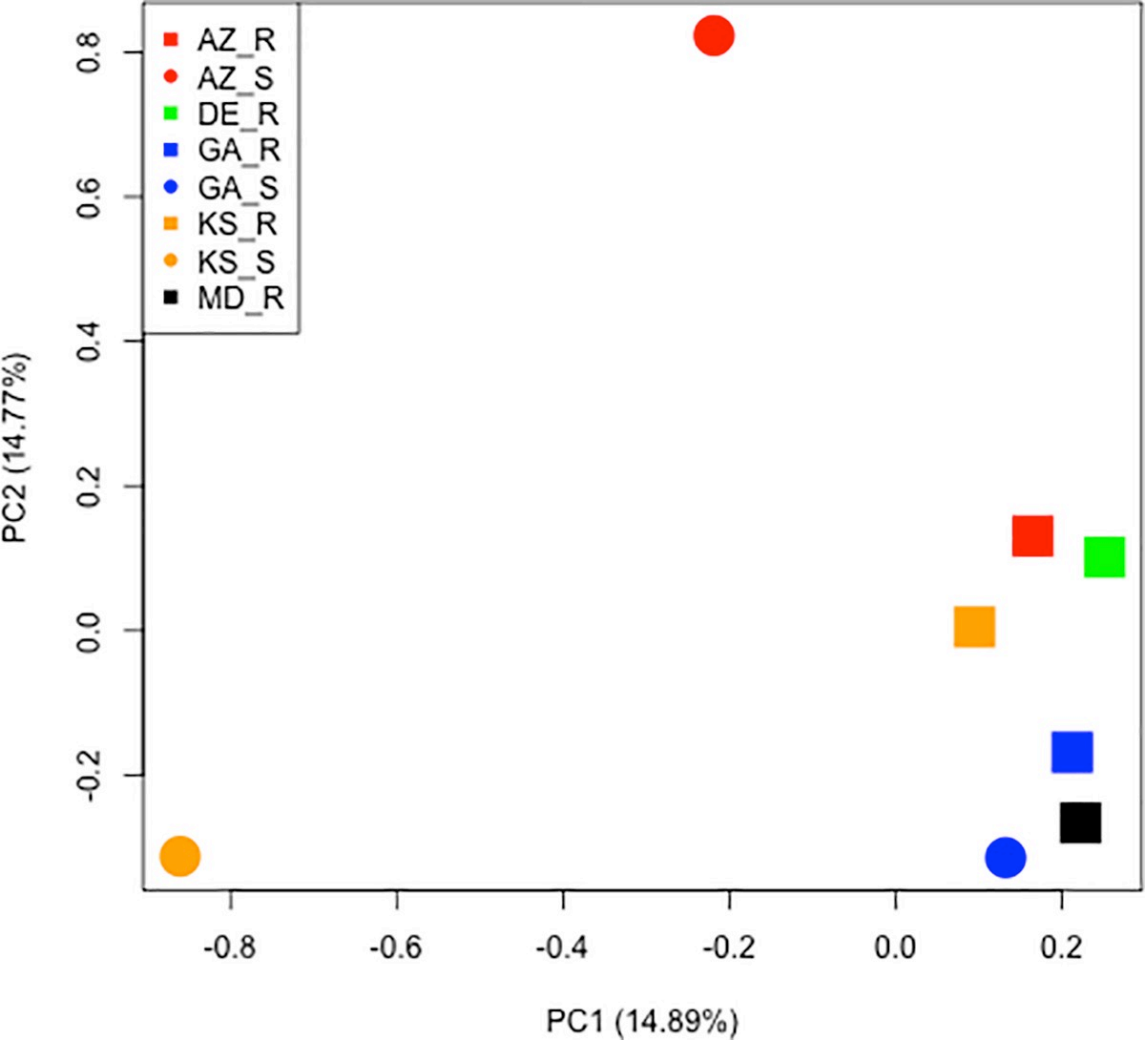
Hierarchal clustering by principal components of genome-wide SNPs. Principal component clustering using ~15k genome-wide SNPs pruned for linkage equilibrium of GR and GS individuals from geographically distant locations.

Reads that did not map to the replicon were aligned to the glyphosate sensitive reference assembly to assess relatedness among the nuclear genomes. We discovered a total of 2,194,814 biallelic SNPs among the 8 samples. Using an LD pruned set of genome-wide SNPs (~15k), hierarchal clustering by principal coordinates accounted for ~29% of the genetic variation in the first two eigen vectors (Fig 6). Glyphosate resistant samples form a distinct cluster around the glyphosate sensitive sample from GA, while AZ and KS sensitive samples are significantly diverse from their resistant matched pair (Fig 6). Clustering of the geographically distant resistant biotypes among each other and the sensitive sample from GA further supports a single origin event from GA and spread by mechanical means through seed. The geographical distance of the AZ and KS sensitive samples from GA source is also supported by genetic analysis in Fig 6. Two surveys of the most troublesome weeds in southern states (1994 and again in 2005) revealed a dramatic shift in weed flora as a result of massive increases in glyphosate use in Roundup Ready soybean, cotton, and corn [29]. The exceptional weed control offered by this new transgenic system increased glyphosate usage and exposure of weed populations to glyphosate; while the use of several other herbicides dramatically decreased. This technology also supported reduced and no tillage production practices. In hindsight, the combined effect provided conditions that may have supported selection for resistance. The relative rank of *A*. *palmeri* rose in these surveys from 25 to 4 in corn, 10 to 2 in cotton and 23 to 2.8 in soybean, which was largely due to glyphosate resistance. The clustering observed in Figs 5 and 6, with conservation of replicon and genomic sequences, respectively, supports the hypothesis that GR biotypes may have initially displaced or supplanted the GS biotypes by movement of seed and pollen, rather than outcrossing directly into eastern populations; whereas GS populations more distant to the west had less displacement or introgression.

Although it cannot be stated that all cases of glyphosate resistance are due to the highly conserved *EPSPS* replicon, the GR samples sequenced here were nearly identical with consistent depth of reads and the exact size and position of components (spikes, genes) across six states with significant geographic separation between the GR biotypes when analyzed with the eccDNA replicon as a reference. Additionally, every gene in the replicon had an identical read depth, indicating that the entire replicon is present and at the same abundance (S2 Table). The read sequences mapped to the *EPSPS* consensus sequence with near perfect concordant and contiguous alignments with similar abundances of gene copies, repeat elements, and low-complexity sequence. The mapped reads compared across replicon sequences from different locations also contained very few polymorphisms (SNPs/INDELS) and read depths were consistent and greater than 1k, which provided further evidence that the replicons are significantly amplified in GR biotypes. Moreover, SNP diversity of the amplified genes has been reduced to singular homologous forms in the *EPSPS* replicon because of expansion by high copy numbers of the singular replicon sequence. Overall, there has been a reduction of genetic variation for the *EPSPS* which also points to the possibility that the replicon and replicon-based resistance appears to be a singular event. Hence, these results support the hypothesis that the replicon originated in one location, possibly from a single source, then rapidly spread across the United States through human or other natural dispersal means.

The replicon not only provides a unique heritable unit enabling adaption to herbicide stress, but also adds another 58 putative genes (aside from *EPSPS*) and gene products which could conceivably alter the genomic trajectory of this species. Glyphosate resistance has not negatively impacted *A*. *palmeri* fitness [8–10] and the additional 41 expressed genes may provide advantages in areas such as abiotic and biotic stress tolerance [11]. Replicon-based glyphosate resistance represents a natural, molecular solution constructed, modified and initiated by *A*. *palmeri*, a consequence that may inform future efforts in biomolecular engineering of genes that promote survival and genomic success.

The replicon can be found both integrated within the nuclear genome and tethered externally to chromosomes indicating the presence of bimodal forms of transmission of the replicon to daughter cells [14]. The mechanisms regulating distribution patterns of replicons in dividing cells is unknown, but may be determined by the number of available tethering sites as well as the number of copies of replicons present in the genome. Inheritance patterns are still poorly understood, but current data supports a non-mendelian inheritance pattern [9, 15–19] that may be driven by disproportionate replicon association in the genome during cell division. This may account for the copy number variations observed in *A*. *palmeri* populations and explains why F_2_ offspring from a resistant and susceptible test cross do not show the predicted 3:1 segregation of resistant: susceptible phenotype [15–19]. This also implies that the amplified *EPSPS* sequence and other genes within the replicon may not be genetically linked with any other trait in the genome and undergo independent assortment from the rest of the genome, which in turn, implies inheritance of the *EPSPS* resistance trait (i.e. the *EPSPS* replicon) does not extort the same amount of linkage drag [30, 31]. Once a resistant plant from a distant location crosses with a locally adapted susceptible plant, the resulting offspring can backcross with the local susceptible plants and purge any negative traits that may have been initially co-introduced with glyphosate resistance.

The origins of the replicon, from its initial stages to its present-day configuration are unknown, but likely occurred in a short evolutionary window. The replicon may have been formed through circularization hotspots [32], or from genome shuffling, reorganization, and recombination events within the genome with influence from mobile genetic elements [33]. Circularization hotspots, while again unknown, may be regions of repetitive sequences capable of intra-molecular recombination. Such regions are common in the replicon. Parts of the *EPSPS* replicon as partial or intermediary forms, or its building blocks, have not been found to be contiguous or in similar abundance in GS plants. The conditions responsible for the selection pressure under which the replicon was initially formed are also unknown. Elucidating the compounded evolutionary events leading to its development, such as chromatin reorganization/genome reshuffling, and evidence for segmental duplications and non-homologous end joining events, would improve our understanding of how such a structure was generated. The added genomic content, whereas it does result in an imbalance in gene dosage, does not appear to rise to a case of aneuploidy because the replicon is much smaller than the entire chromosome, lacks a centromere, and it exhibits nonmendelian genetics. Finally, due to the conservation of the nucleotide content and structure of the *EPSPS* replicon in the geographically diverse populations used in this study, the results strongly suggest that the events that gave rise to this form of glyphosate resistance likely only occurred once.

## Supporting information

S1 Table Read depths for each wgs dataset aligned to the eccDNA replicon. Number of whole genome shotgun reads that align to each nucleotide of the eccDNA replicon reference assembly. Average read depths are in parenthesis.
(XLSX)

S2 Table CNVnator read depths of putative CNVs. This table presents relative read depths as determined by CNVnator for each gene in the *Amaranthus palmeri* and eccDNA replicon reference assemblies. A gene is considered duplicated when relative read depths are greater than 2.
(XLSX)

S3 Table SNPs by nucleotide position in the cassette. The REF column is the eccDNA replicon reference allele. X/X indicates the plant is homozygous for the reference. X/YYY indicates there is an insertion../. Indicates the genotype is missing (no reads at this position).
(XLSX)
